# Helical tomotherapy to LINAC plan conversion utilizing RayStation Fallback planning

**DOI:** 10.1002/acm2.12032

**Published:** 2017-01-19

**Authors:** Xin Zhang, Jose Penagaricano, Ganesh Narayanasamy, Peter Corry, TianXiao Liu, Maraboyina Sanjay, Nava Paudel, Steven Morrill

**Affiliations:** ^1^ Radiation Oncology Department University of Arkansas for Medical Sciences Little Rock AR 72205 USA; ^2^ Radiation Oncology Department Houston Methodist Cancer Center Sugar Land TX 77479 USA

**Keywords:** dosimetric comparison, Fallback treatment planning, helical tomotherapy, RayStation

## Abstract

RaySearch RayStation Fallback (FB) planning module can generate an equivalent backup radiotherapy treatment plan facilitating treatment on other linear accelerators. FB plans were generated from the RayStation FB module by simulating the original plan target and organ at risk (OAR) dose distribution and delivered in various backup linear accelerators. In this study, helical tomotherapy (HT) backup plans used in Varian TrueBeam linear accelerator were generated with the RayStation FB module. About 30 patients, 10 with lung cancer, 10 with head and neck (HN) cancer, and 10 with prostate cancer, who were treated with HT, were included in this study. Intensity‐modulated radiotherapy Fallback plans (FB‐IMRT) were generated for all patients, and three‐dimensional conformal radiotherapy Fallback plans (FB‐3D) were only generated for lung cancer patients. Dosimetric comparison study evaluated FB plans based on dose coverage to 95% of the PTV volume (R_95_), PTV mean dose (D_*mean*_), *Paddick's* conformity index (CI), and dose homogeneity index (HI). The evaluation results showed that all IMRT plans were statistically comparable between HT and FB‐IMRT plans except that PTV HI was worse in prostate, and PTV R_95_ and HI were worse in HN multitarget plans for FB‐IMRT plans. For 3D lung cancer plans, only the PTV R_95_ was statistically comparable between HT and FB‐3D plans, PTV D_mean_ was higher, and CI and HI were worse compared to HT plans. The FB plans using a TrueBeam linear accelerator generally offer better OAR sparing compared to HT plans for all the patients. In this study, all cases of FB‐IMRT plans and 9/10 cases of FB‐3D plans were clinically acceptable without further modification and optimization once the FB plans were generated. However, the statistical differences between HT and FB‐IMRT/3D plans might not be of any clinically significant. One FB‐3D plan failed to simulate the original plan without further optimization.

## 1 Introduction

Generally, treatment planning software system (TPS) is an integrated software package that allows the target and organs at risk (OAR) definitions, management of treatment plan, plan optimization, and delivery quality assurance (DQA). It also includes the DICOM import and export and data management system application software for archiving and management of patient data. TPS such as Eclipse (Varian Medical Systems, Palo Alto, CA, USA), Tomotherapy (Accuracy Inc, Sunnyvale, CA, USA), Pinnacle (Philips Healthcare, Andover, MA, USA), RayStation (RaySearch Medical Laboratories, Stockholm, Sweden) have different dose calculation engines as well as other characteristics that are unique to each system. Furthermore, each TPS needs to be commissioned using beam data from the linear accelerator to be used for patient treatment delivery. For example, a treatment plan generated from TPS that is commissioned to Varian Clinac iX linear accelerator could not be directly used to treat with Varian TrueBeam linear accelerator. In summary, there is no easy way to transfer patient treatment plans between different TPSs without repeating a significant amount of work.

Due to the lack of interchangeability among TPSs, there is a need to develop a method that can automatically transfer patient plans from one treatment unit/TPS to another treatment unit/TPS. This is especially useful for treatment centers that have multiple treatment units and TPSs that want to switch patients due to, for example, scheduling conflicts and machine down time.

Recently, RayStation TPS developed several advanced features to generate backup treatment plans.^1^ RayStation TPS has a module named Fallback (FB) which uses a dose mimicking technique to create a backup plan, enabling a patient to be treated on another machine, possibly with a different treatment technique. At present and to our knowledge, there is no dosimetric evaluation published in the literature for the RayStation FB module. The purpose of this study was to provide insight into the use of the RayStation FB module for generating 3D and IMRT FB backup plans (FB‐IMRT and FB‐3D plans) that can be delivered at different treatment machines. Helical tomotherapy (Accuracy Inc, Sunnyvale, CA, USA) is a treatment machine that is heavily used in our clinic; we select the HT as the original treatment machine and Varian TrueBeam STx linear accelerator (Varian Medical Systems, Palo Alto, CA, USA) as a backup machine according to our clinical setup. HT backup plan using RayStation Fallback module was generated and compared with the original HT plan. An additional goal was to evaluate the dosimetric equivalency between the original HT and the FB plans.

## 2 Methods and materials

### 2.A Patient characteristics

Thirty previously treated patients using HT were selected for this study including 10 lung cancer patients, 10 HN cancer patients, and 10 prostate cancer patients. These treatment sites were selected as representing typical treatment sites in most clinics. The prescription dose was at least 95% of the PTV to receive 60 Gy in 8 fractions for lung cancer patients, 66 Gy in 30 fractions for HN patients, and 54 Gy in 30 fractions for prostate cancer patients. In the prostate cases, only the initial plan to 54 Gy was used and the boost plans were not included in this study. All FB plans were generated using RayStation FB module (v.4.5.1.14) and using the same prescription doses as the original HT treatment plans. All the original HT plans are intensity‐modulated radiotherapy treatment plans.

Each patient's original DICOM RT images, DICOM RT structures, DICOM RT plan, and DICOM RT dose files were transferred from HT TPS to the RayStation TPS. Before each Varian linear accelerator FB plan is generated, the CT density table dedicated to RayStation TPS has to be selected manually. A detail flow diagram of how a treatment plan conversion from one treatment unit/TPS to another treatment unit/RayStation is showed in the following Fallback module flow diagram.

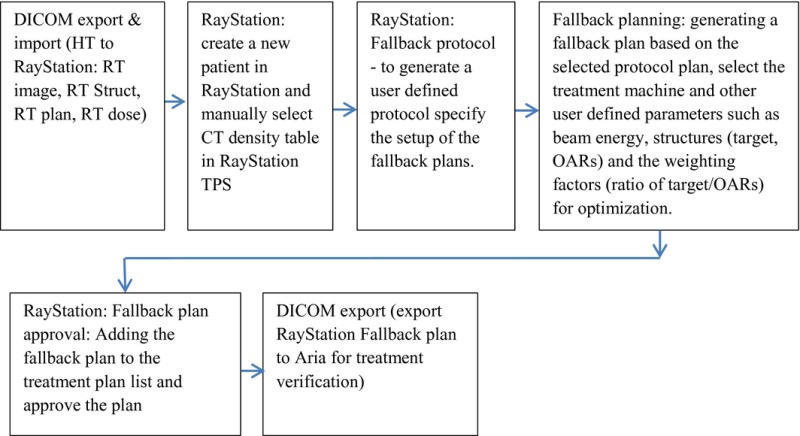

### 2.B Fallback protocol creation

A specific protocol plan needs to be pregenerated before creating any replacement FB plan to specify the setup of the Fallback plans. A FB plan was created by extracting information from a protocol plan generated using the FB module in RayStation TPS. The extracted information includes treatment planning parameters such as treatment techniques (3D, IMRT, or VMAT), beam geometry (gantry, collimator, couch, and other accessory settings), optimization parameters such as weighting factor of dose mimic between the target and the organ at risk (OARs). These parameters can be edited by the user and it is possible to test the FB protocol plan by using the dose mimicking technique to compare the FB plan and the original HT plan using a number of visual tools (i.e., dose volume histogram (DVH) curves, dose differences).

The precision of FB plan dose simulation is greatly related to the pregenerated protocol plan. The protocol plans can be used as a shared protocol plans such as tumor‐specific protocol plans (lung, HN, prostate), treatment technique‐specific protocol plans (IMRT, 3D, VMAT), energy‐specific protocol plans (6 MV, 10 MV), beam angle‐specific protocol plans (i.e., six field, seven field, or nine field), target position‐specific protocol plans (i.e., head first or feet first). The protocol plans also can be very specific used as a patient‐specific protocol plan. A more specific protocol plan will result in a much higher degree of correspondence between the original HT plan and the resultant FB plan; however, a great deal of time and effort will be needed to generate these protocol plans.

### 2.C Fallback plan creation

In this study, lung and HN IMRT FB plans shared the same single protocol plan for each patient with head first supine position and prostate IMRT FB plans shared another single protocol plan for each patient with feet first supine position. The protocol plan parameters used for all FB‐IMRT plans included: nine field beams with fixed gantry angles of 40, 80, 120, 160, 200, 240, 280, 320, and 360 degrees; collimator angle of 0 degree; couch angle of 0 degree; and a static multileaf collimator (sMLC). The FB plan use dose mimicking optimization algorithm to optimize the Fallback plan. The goal of the dose mimicking optimization is to minimize the error in DVH between the reference plan (original HT plan) and the deliverable plan (Linac Fallback plan). Functions associated with OARs and targets are given a weighting factor equal to a user‐defined target priority (Target/OARs ratios). In this study, the dose mimicking target/OAR optimization weighting factor was set to 100.00 which means the importance of the optimization goal for target over OARs is 100. Usually, the higher the ratio, the more importance for the target dose simulation and the lower the ratio, the more importance for the OARs dose simulation.

The energies of 6 MV were selected for lung and HN patients and 10 MV was selected for prostate patients. For FB‐3D plans, patient‐specific individual protocol plans were used. The plan parameters such as gantry, collimator, couch, and wedge angles for the FB‐3D plans were determined individually and the final protocols selected were the ones that could best mimic the original HT plans.

For lung cancer patients, both FB‐3D and FB‐IMRT plans were evaluated. For HN and prostate cancer patients, only FB‐IMRT plans were evaluated because IMRT treatment technique is the most commonly used treatment technique for HN and prostate cancer patients.

The quantitative evaluation of PTV dose distribution included: mean dose of PTV (*D*_*mean*_), the PTV dose coverage R_95_ ($R_{95}=\frac{D_{95\%}}{D_{prescription}}$) where D_*x%*_ is the dose to *x%* of the target volume, *Paddick's* conformity index (CI)^2^, and homogeneity index (*HI*). *CI* was defined by the following equation.

(1) $$CI=\frac{TV_{PIV}^{2}}{TV\times V_{PIV}}$$

Where *TV* is the target volume, *TV*_*PIV*_ is the target volume covered by the prescription isodose volume (PIV), and *V*_*PIV*_ is the total prescription isodose volume. *HI* was defined by the following equation

(2) $$\text{HI}=\frac{D_{2\%}-D_{98\%}}{D_{\mathrm{prescription}}}\times 100\%$$

Dosimetric data comparison between the FB plan and the original HT plan was performed using Wilcoxon matched‐pair signed rank test to clarify the differences in our results. Values of *P ≤ 0.05* were considered significant.^3^

### 2.D FB treatment plan dose verification

The FB plan dose and deliverable verification using Varian TrueBeam linear accelerator was verified by patient‐specific quality assurance (QA). The QA was performed using ArcCheck diode array (ArcCHECK, Sun Nuclear Corporation, Melbourne, USA). Measured data were generally compared against planning data using two dimensional (2D) gamma analyses with percent dose difference (%DD) and distance‐to‐agreement (DTA) criteria. The analysis was performed in SNC Patient software version 6.2.1 (Sun Nuclear, Melbourne, FL, USA) using 3%/3 mm as dosimetric difference and distance‐to‐agreement criteria. A 10% dose threshold and global normalization was used.

## 3 Results

**Figure 1:**
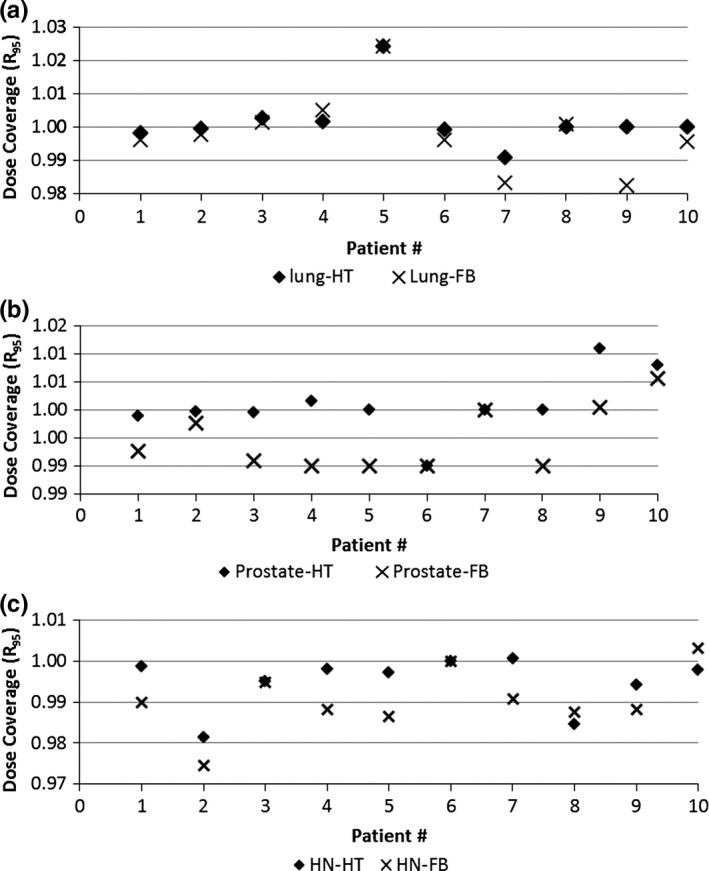
(a–c) The dose coverage R_95_ from FB‐IMRT and HT plans for lung (1a), prostate (1b), and HN (1c) patients.

**Figure 2:**
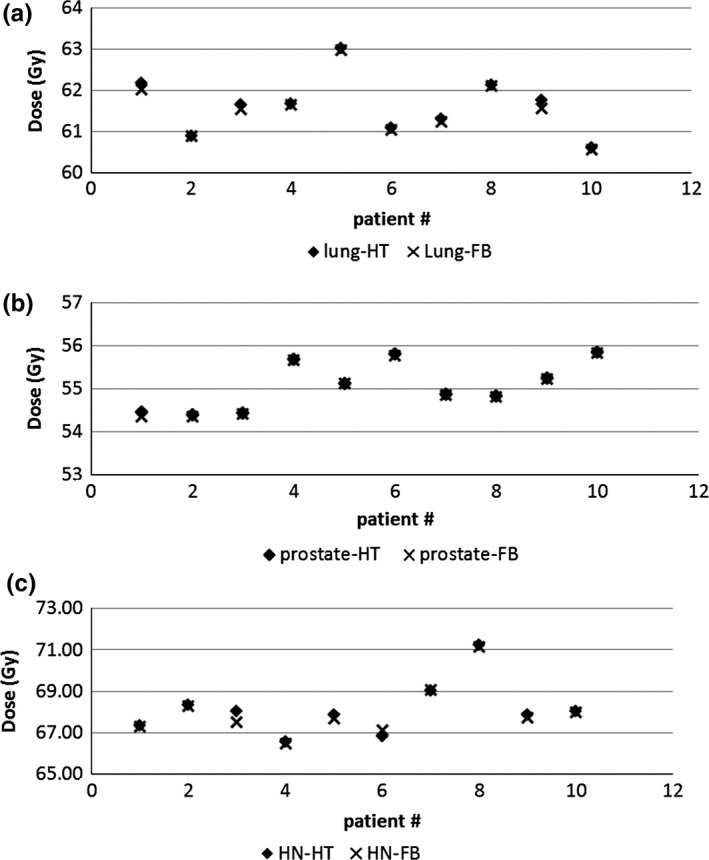
(a–c) PTV D_mean_ from FB‐IMRT and HT plans for lung (2a), prostate (2b), and HN (2c) patients.

**Figure 3:**
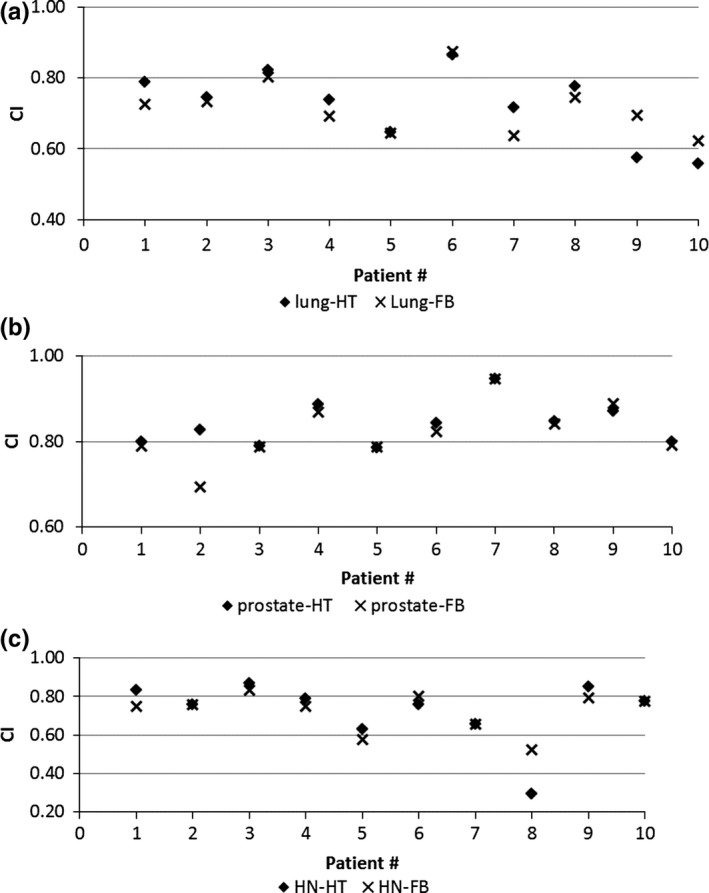
(a–c) PTV CI from FB‐IMRT and HT plans for lung (3a), prostate (3b), and HN (3c) patients.

**Figure 4:**
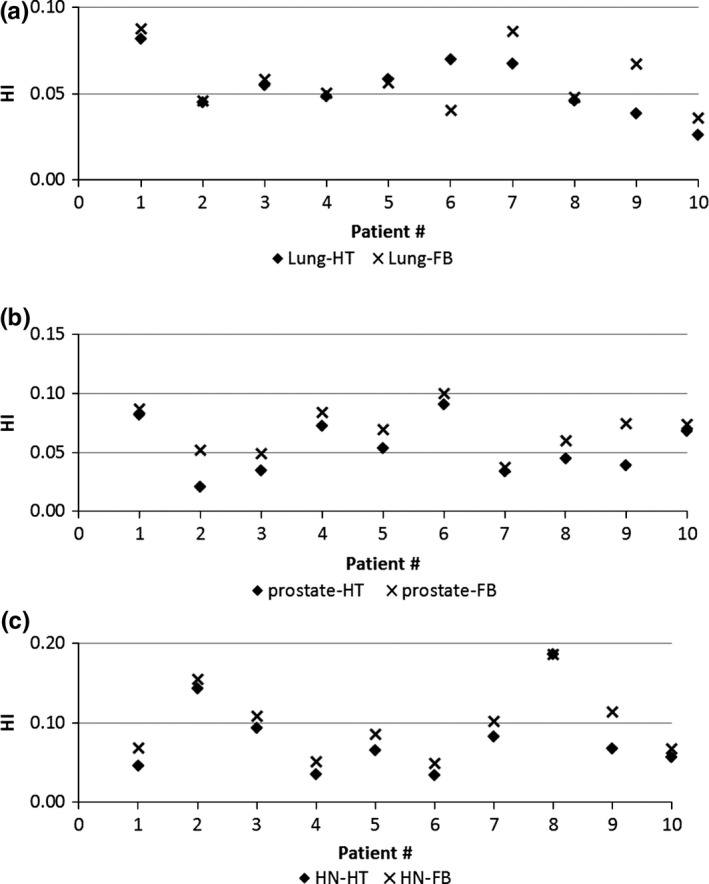
(a–c) PTV HI from FB‐IMRT and HT plans for lung (4a), prostate (4b), and HN (4c) patients.

The PTV dose coverage R_*95*_, D_*mean*_, CI, and HI from FB‐IMRT and HT plans for lung, prostate, and HN patients are shown in Figs. 1(a)–(c), 2(a)–(c), 3(a)–(c), and 4(a)–(c), respectively.

Figures 1(a)–(c) show that all the FB‐IMRT plans satisfied the prescription dose of at least 95% of the PTV to receive the prescription dose. The median values and ranges of PTV D_mean,_ R_95_, CI, and HI from FB‐IMRT plans and HT plans for all the patients are listed in Table 1. A Wilcoxon matched‐pair signed rank test showed no statistical difference for PTV R_95_, CI, and HI, whereas there is statistical difference for D_mean_ between FB‐IMRT and the original HT plans for lung cancer patients. The median mean dose difference was 0.11 Gy between FB‐IMRT and HT plans. For HN patients, there was no statistically significant difference for PTV D_mean_ and CI, whereas there were statistical differences for PTV R_95_ and HI which were worse in FB‐IMRT plans. For prostate patients, there were no statistical differences for PTV R_95_, D_mean_, and CI, whereas there was a statistical difference for HI which was worse for FB‐IMRT plans compared to HT plans.

**Table 1:** Median and ranges for PTV D_mean_, R_*95*_, CI, HI from FB‐IMRT and HT plans for lung, HN, and prostate patients

		D_mean_ (Gy)	R_95_	CI	HI
Lung	HT	61.68 (60.61–63.03)	1.00 (0.99–1.02)	0.74 (0.56–0.86)	0.052 (0.03–0.08)
	FB‐IMRT	61.57 (60.57–62.99)	0.99 (0.98–1.02)	0.71 (0.62–0.88)	0.053 (0.04–0.09)
	Significant?	Yes	No	No	No
HN	HT	67.95 (66.57–70.12)	1.00 (0.98–1.00)	0.78 (0.63–0.87)	0.060 (0.03–0.13)
	FB‐IMRT	67.71 (66.5–70.05)	0.99 (0.97–1.00)	0.75 (0.58–0.83)	0.077 (0.05–0.16)
	Significant?	No	Yes	No	Yes
Prostate	HT	55.01 (54.41–55.85)	1.00 (0.99–1.01)	0.84 (0.79–0.95)	0.049 (0.02–0.09)
	FB‐IMRT	55.00 (54.37–55.83)	1.00 (0.99–1.01)	0.84 (0.79–0.97)	0.064 (0.04–0.099)
	Significant?	No	No	No	Yes

The global PTV maximum dose differences between the HT and FB‐IMRT plans were also evaluated and the median maximum dose differences between HT and FB‐IMRT plans were 0.22 Gy with the range of 0.03 Gy to 1.00 Gy for lung patients, 0.88 Gy with dose range of 0.17 Gy to 1.38 Gy for prostate patients, and 0.95 Gy with dose range of 0.28 Gy to 2.83 Gy for head and neck patients.

The median and range of OAR doses for FB‐IMRT and HT plans for lung, HN, and prostate patients are listed in Table 2(a–c). For lung cancer patients, there was no statistically significant difference in cord dose and there were statistically significant difference for all the other OARs (*P* < 0.05) which received higher doses in HT plans. For the prostate patients, there were statistically significant differences for all OARs doses which received higher doses in HT plans. For the HN patients, there was a statistically significant difference for the cord and larynx dose, where cord dose was lower and larynx dose was higher in HT plans compared to FB‐IMRT plans and there were no statistically significant differences for all the other OAR doses between FB‐IMRT plans and HT plans.

**Table 2:** Median and ranges for OAR doses from FB‐IMRT and HT plans for (a) lung patients, (b) prostate patients, (c) HN patients

	HT	FB‐IMRT	Difference?
(a) Lung OARs (Gy)			
Cord (D_max_)	22.53 (8.19–38.92)	24.49 (7.86‐38.17)	No
Lung (normal)	6.33 (6.03–21.44)	5.83 (5.4–19.12)	Yes
Heart	2.58 (0.34–26.34)	2.39 (0.07–24.92)	Yes
Esophagus	9.36 (2.32–34.61)	9.01 (2.24–34.61)	Yes
Body	3.86 (1.72–10.35)	3.51 (1.45–9.59)	Yes
(b) Prostate OARs (Gy)			
Bladder	32.04 (20.73–48.92)	31.13 (18.96–48.13)	Yes
Rectum	25.675 (20.16–40.54)	26.675 (20.46–42.29)	Yes
Femur head (R)	9.96 (6.65–12.92)	8.64 (5.6–10.68)	Yes
Femur head (L)	10.23 (6.47–14.44)	8.89 (5.35–12.54)	Yes
Body	3.85 (3.2–6.65)	3.36 (2.88–5.79)	Yes
(c) HN OARs (Gy)			
Cord (D_max_)	41.37 (12.25–52.88)	44.78 (16.34–57.19)	Yes
R Parotid	27.27 (0.35–41.9)	27.93 (0.26–38.12)	No
L Parotid	27.22 (0.51–43.54)	27.23 (0.22–41.36)	No
Larynx	42.98 (15.7–50.04)	41.45 (16.1–49.29)	Yes
Body	11.29 (1.7–21.51)	10.88 (1.75–20.64)	No

The maximum dose differences for OARs between the FB‐IMRT and HT plans were also evaluated and the median maximum dose differences were less than 1Gy for all the OARs in the treatment plan conversion between HT plan to FB‐IMRT plans for all the lung, HN, and prostate patients.

The comparison between the FB‐3D plan and the original HT plan was performed for lung cancer patients. The median values and ranges for PTV D_*mean*_, R_95_, CI, and HI for total nine lung patients (1/10 patients was excluded because of an unacceptable treatment plan) are listed in Table 3(a). A Wilcoxon matched‐pair signed rank test showed there were statistically significant differences for PTV D_mean_, CI, and HI, whereas there was no statistically significant difference for PTV R_95_ between FB‐3D and HT plans. The FB‐3D plan has higher PTV D_mean_ and worse CI and HI compared to the original HT plan.

**Table 3:** Median and ranges for (a) PTV D_mean_, R_*95*_, CI, HI, (b) OAR doses from FB‐3D and HT plans for lung patients

	FB‐3D	HT	Difference?
(a) PTV			
D_mean_ (Gy)	62.61 (61.23–64.26)	61.69 (60.61–63.03)	Yes
R_95_	1.00 (0.95–1.00)	1.00 (0.99–1.02)	No
CI	0.46 (0.28–0.67)	0.74 (0.56–0.86)	Yes
HI	0.1 (0.04–0.18)	0.048 (0.03–0.08)	Yes
(b) OAR Doses (Gy)			
Cord (D_max_)	24.33 (10.64–37.85)	21.3 (9.38–38.92)	No
Lung (normal)	5.67 (3.74–20.27)	6.13 (4.11–21.43)	Yes
Heart	1.32 (0.05–17.32)	1.95 (0.34–16.12)	No
Esophagus	6.87 (1.12–28.04)	9.26 (1.42–27.83)	No
Body	2.46 (1.47–8.11)	3.07 (1.72–8.27)	Yes

The median values and ranges of OAR doses calculated from the FB‐3D plans and HT plans for lung patients were listed in Table 3(b). A Wilcoxon matched‐pair signed rank test showed statistically significant difference for normal lung and body and HT plans received higher doses compared to FB‐3D plans. There were no statistical differences for all other OAR doses.

Fallback plan QAs were performed and the results showed that the mean ± standard deviation of gamma agreement index score was 98% ± 1% and higher (3%, 3 mm criteria) for lung, HN, and prostate patients. These QA results also showed that the FB plans are clinically equivalent to the original HT plans and can be successfully delivered on the backup Varian TrueBeam accelerator.

## 4 Discussion

FB‐IMRT and FB‐3D plans were generated in RayStation Fallback module based on the pregenerated protocols. No extra efforts were made to improve these FB plans once the protocol plan was adhered to. The evaluation of FB plans was performed by comparing the dosimetric parameters calculated from FB plans and the original HT plans for the selected HN, lung, and prostate cancer patients. These patients were specifically selected to represent typical IMRT treatment plans at different anatomic locations.

For lung cancer patients, FB‐IMRT plans successfully simulated the original HT plan. The PTV doses coverage R_95_, CI, and HI were all comparable between HT plans and FB‐IMRT plans. The statistical comparison showed PTV D_mean_ was higher in HT plans; however, the dose difference was only 0.11 Gy and it is clinically insignificant. The OAR doses were generally lower calculated from FB‐IMRT plans compared to HT plans. This is mainly due to the nature of the HT beams with which the dose is delivered in a helical fashion with 51 projections around the PTV target,^4^, ^5^ whereas only static beams were delivered in LINAC Fallback plans. For the cord dose, HT plans used higher importance weighting factors during the plan optimization which makes it comparable with that in FB‐IMRT plans. However, these OAR differences might not be of any clinically significant.

For prostate patients, the FB‐IMRT plans also successfully simulated the original HT plans. The PTV dose coverage R_95_, D_mean_, and CI were comparable between FB‐IMRT plans and HT plans. The HI was worse for the FB‐IMRT as compared to HT plans. However, the maximum HI value difference of 0.02 makes it clinical insignificance. For the OAR doses, prostate patients also received higher OAR doses from HT plans compared to FB‐IMRT plans as mentioned previously.

**Figure 5:**
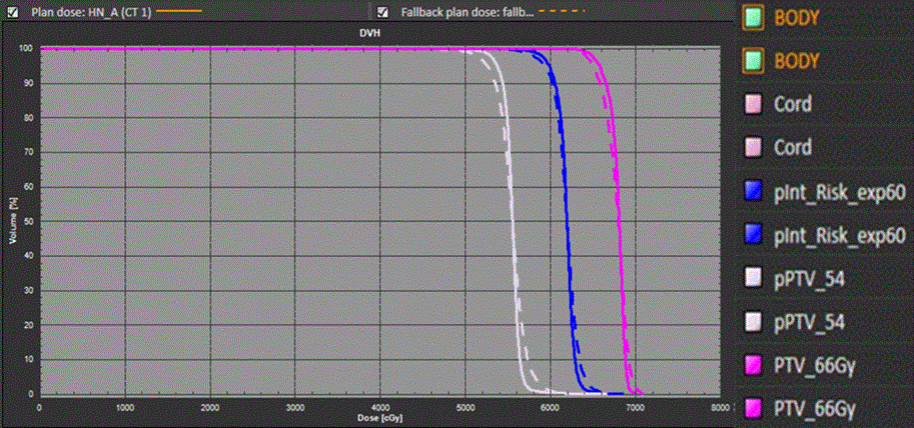
Example of DVH calculated from FB‐IMRT and HT plans with three targets (PTV66, PTV‐60, and PTV54 and Target/OARs optimization weighting factor = 100) for one HN patient.

**Figure 6:**
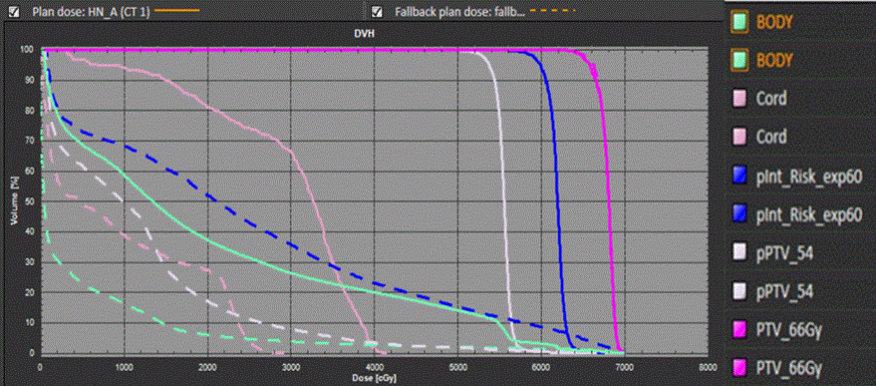
Example of DVH calculated from FB‐IMRT and HT plans with one PTV target (PTV66 and Target/OARs optimization weighting factor = 100) for one HN patient.

For HN patients, the PTV D_mean_ and CI were comparable between FB‐IMRT plans and HT plans, whereas the PTV coverage R_95_ and HI were worse for FB‐IMRT plans compared to the HT plans. This could be explained by the following reason. For HN patients, multiple targets in the original HT plans (PTV‐66 Gy, PTV‐60, and PTV‐54) were used and PTV‐66 represented the high‐risk volume which included all regions with gross disease as seen on diagnostic CT; PTV‐60 was the intermediate risk volume which included regions with a suspicion of microscopic disease; PTV‐54 was the low‐risk volume which included regions receiving prophylactic treatment.^6^ The selection of multiple targets has significantly affected the dose simulation results for FB treatment plans. Figs. 5 and 6 showed FB‐IMRT dose simulation results (PTV‐66, PTV‐60, PTV‐54, cord, body) for one of the representative HN patients.

We noticed that only the DVH of PTV‐66 had an acceptable agreement between FB‐IMRT and original HT plans comparing single‐target dose simulation (Fig. 6) and multitarget dose simulation (Fig. 5). PTV‐60 and PTV‐54 failed to mimic the original HT plan target doses in Fig. 6 because these two targets were not included in the target dose simulation. Cord dose was much smaller using single‐target dose simulation (D_max_ = 29.35) vs. the multitarget dose simulation (D_max_ = 45.49 Gy). Thus, for multitarget treatment plan, all the targets need to be included to generate an acceptable Fallback plan. FB plan simulated the PTV and OAR dose better for single‐target original plan compared to multitarget original plan and this can also explain the DVH dose simulation differences between the HN patients (multitargets) and lung and prostate cancer patients (single target).

The comparison between HT plans and FB‐3D plans was also evaluated for lung cancer patients. We only evaluated FB‐3D plans for lung cancer patients because IMRT treatment technique is the most commonly used treatment technique for prostate and HN cancer patients. This study showed that one patient's FB‐3D plan was not simulated successfully when compared to the original HT plan with either the PTV or the cord dose being too high to be acceptable. For the failed treatment plan, different target/OAR optimization weighting ratios were tested and dose simulation results showed that either the PTV dose or the cord maximum dose was too high to be clinically acceptable. For a smaller target/OAR optimization weighting ratio (weighting ratio = 1), a FB plan was obtained with PTV D_mean_ = 71.77 Gy and cord D_max_ = 49.6 Gy. On the other hand, for a larger target/OAR optimization weighting ratio (weighting ratio = 1000), a FB plan was obtained with PTV D_mean_ = 66.73 Gy, cord D_max_ = 62.65 Gy. Thus, both the FB plans were clinically unacceptable. This study indicates that the value of target/OAR optimization weighting ratio is directly related to the dose simulation results for both PTV and OAR doses. In general, a higher value of target/OAR optimization weighting ratio corresponds to better PTV dose coverage and lower ratio corresponds to better OAR dose sparing. Note that only nine lung patient FB‐3D plans were used for the dosimetric comparison with HT lung plans in this study. The statistical comparison results showed that while PTV R_95_ was comparable between HT plans and FB‐3D plans, the PTV D_mean_ was higher for FB‐3D plans and CI and HI were worse compared to HT plans. These results were expected as HT is an IMRT treatment plan and it is a high‐precision technique especially on PTV conformity and homogeneity compared to 3D treatment technique.^7^ This study shows that FB‐3D plan could be an easy replacement backup plan for those cases where IMRT is not an available option. In addition, the FB‐3D plans could be improved in RayStation TPS if needed.

## 5 Conclusions

Helical tomotherapy backup plans used in Varian TrueBeam linear accelerator were generated using RayStation Fallback module and the FB plans and the original HT plans were compared. The Fallback plans were generated based on the preselected protocol plans and no further optimization and modification were performed to improve the FB plan as long as the protocol plan was selected in this study.

All the FB‐IMRT plans were acceptable for use in clinic. There were some statistical differences when comparing different types of FB‐IMRT plans with the original HT plans; however, these differences might not be of any clinically significant: lung FB‐IMRT plans had comparable PTV R_95_, CI, and HI; prostate FB‐IMRT plans had comparable R_95_, D_mean_, CI, and worse HI compared to the original HT plans and HN FB‐IMRT plans had comparable D_mean_, CI, and worse R_95_, HI compared to the original HT plans.

FB‐3D plans were also generated in RayStation TPS for lung cancer patients and it had higher D_mean_ and worse CI and HI compared with the original HT plans in this study. It was noted that FB‐3D plans could fail to simulate doses from the original HT plan and might require more time and effort to create an acceptable plans compared to FB‐IMRT plans.

## Conflict of Interest

The authors declare no conflict of interest.
